# Effects of dielectric permittivities on skin heating due to millimeter wave exposure

**DOI:** 10.1186/1475-925X-8-20

**Published:** 2009-09-23

**Authors:** Akio Kanezaki, Akimasa Hirata, Soichi Watanabe, Hiroshi Shirai

**Affiliations:** 1 Chuo University, Department of Electrical, Electronic, and Communication Engineering, Tokyo, Japan; 2 National Institute of Information and Communications Technology, EMC group, Tokyo, Japan; 3 Nagoya Institute of Technology, Nagoya, Department of Computer Science and Engineering, Japan

## Abstract

**Background:**

Because the possibility of millimeter wave (MMW) exposure has increased, public concern about the health issues due to electromagnetic radiation has also increased. While many studies have been conducted for MMW exposure, the effect of dielectric permittivities on skin heating in multilayer/heterogeneous human-body models have not been adequately investigated. This is partly due to the fact that a detailed investigation of skin heating in a multilayer model by computational methods is difficult since many parameters are involved. In the present study, therefore, theoretical analyses were conducted to investigate the relationship between dielectric permittivities and MMW-induced skin heating in a one-dimensional three-layer model (skin, fat, and muscle).

**Methods:**

Approximate expressions were derived for the temperature elevation and temperature difference in the skin due to MMW exposure from analytical solutions for the temperature distribution. First, the power absorption distribution was approximated from the analytical solution for a one-layer model (skin only). Then, the analytical expression of the temperature in the three-layer model was simplified on the basis of the proposal in our previous study. By examining the approximate expressions, the dominant term influencing skin heating was clarified to identify the effects of the dielectric permittivities. Finally, the effects of dielectric permittivities were clarified by applying partial differentiation to the derived dominant term.

**Results:**

Skin heating can be characterized by the parameters associated with the dielectric permittivities, independently of morphological and thermal parameters. With the derived expressions, it was first clarified that skin heating correlates with the total power absorbed in the skin rather than the specific absorption rate (SAR) at the skin surface or the incident power density. Using Debye-type expression we next investigated the effect of frequency dispersion on the complex relative permittivity of tissue. The parametric study on the total power absorbed in the skin showed that skin heating increases as the static permittivity and static conductivity decrease. In addition, the maximum temperature elevation on the body surface was approximately 1.6 times that of the minimum case. This difference is smaller than the difference caused by the thermal and morphological parameters reported in our previous study.

**Conclusion:**

This paper analytically clarified the effects of dielectric permittivities on the thermally steady state temperature elevation and the temperature difference in the skin of a one-dimensional three-layer model due to MMW exposure.

## Background

Millimeter waves (MMW: 30-300 GHz) have become one of the attractive communication tools for short-range and high-capacity transmission due to the manufacturing technology advancement of electronic devices. As MMW usage expands, public concern over human exposure rises, and so it has become important to evaluate human safety regarding MMWs. Most MMW power is absorbed near the surface of the human body, leading to a localized temperature elevation near the surface. There have been a few studies on the relationship between the warm sensation and temperature elevation due to MMW exposure. Three general hypotheses of an adequate stimulus for the warm sensation exist, as summarized by Riu *et al*. [1]: (1) to reach the threshold, the stimulus must produce a fixed temperature elevation in the vicinity of receptors located 150-200 μm below the skin surface [2], (2) a stimulus reaches the threshold when it establishes a fixed temperature difference between two layers located at a depth of approximately 200 and 1,000 μm [3], and (3) a stimulus produces a threshold sensation when the temperature elevation at the receptor layer reaches a level that varies with the concurrent level of adaptation [4]. In addition, the rate of temperature elevation could be another factor. However, the determinative factor of warm sensations is unclear. Therefore, a detailed investigation of the temperature elevation and temperature difference near the body surface is required.

Temperature elevation in human tissues due to electromagnetic wave exposure may be estimated from a bioheat equation (BHE) [5,6]. On the basis of this equation, the mechanism of warm sensation due to microwave exposure has been discussed by Riu *et al*. [1] and Foster *et al*. [7]. They indicated that the body surface temperature elevation in a one-layer model increases with the frequency of the electromagnetic wave. In the MMW region, computational and experimental work has been conducted by Alekseev *et al*. [8-11]. In [8], they proposed a temperature estimation method by solving a conventional BHE as well as a hybrid bioheat equation (HBHE). HBHE combines the conventional BHE and a scalar effective thermal conductivity equation. Comparing the MMW-induced temperature elevation in the skin with the measured one, the effectiveness of HBHE was shown for low and high perfusion rates. In [11], the temperature elevation in a one-dimensional four-layer model was investigated with HBHE.

In our previous study [12], the effect of thermal parameters on the temperature elevation in a three-layer model (skin, fat, muscle) due to MMW exposure was investigated by deriving the analytical solution and its approximation. The main findings in that study were as follows: (1) thermal analysis for a multilayer structure is required even in the MMW region because the temperature elevations for the three-layer model are 1.3-2.8 times larger than those for a one-layer (skin) model and (2) the temperature elevation on the body surface decreases monotonically with the increase of the heat transfer coefficient, terms associated with the blood perfusion, and thermal conductivity.

There have been a few studies on the dielectric permittivities in the MMW region. The variation of the dielectric permittivities of biological tissues [9,13,14] can reach 35% in the MMW region. However, the effect of the dielectric permittivities on tissue heating in the multilayer model has not been adequately investigated. Moreover, the effect cannot be properly evaluated by our previous approximation [12] because those expressions were derived for the temperature elevation at the body surface only, and the parameters associated with the dielectric permittivities are not independent of the thermal and morphological parameters". For these reasons, additional investigations are required to characterize the dependence of MMW-induced skin heating on the dielectric permittivities.

In the present study, theoretical analyses were conducted for the thermal steady state temperature elevation in order to investigate the relationship between the dielectric permittivities and skin heating in a three-layer model. Continuous MMW was assumed to be incident normal to the body surface as a typical worst-case scenario.

## Methods

### Analytical model and approximation for body surface temperature elevation

**Figure 1:**
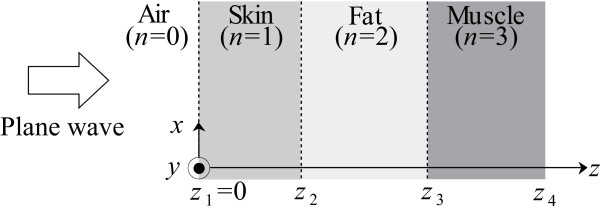
**Thermal constants and tissue thicknesses in the analytical model ("max"-"typical"-"min")**. This model consists of skin (*n *= 1), fat (*n *= 2), and muscle (*n *= 3). The thicknesses of each layer are listed in Table 1.

The model, fundamental solution and approximation methods used in our previous study [12] are also used in the present study. A one-dimensional analytical human body model that consists of skin (*n *= 1), fat (*n *= 2), and muscle (*n *= 3) is shown in Fig. 1. A continuous plane wave is incident normal to the skin layer. The temperature distribution can be derived using the BHE, which is expressed for the one-dimensional model as:

(1)

where *E*(z) is the root-mean-square electric field [V/m], *σ*_*n *_is the electric conductivity [S/m], *κ*_*n *_is the thermal conductivity [W/m °C], *b*_*n *_is the term associated with blood perfusion [W/m^3 ^°C], *A*_*n *_is the metabolic heat production [W/m^3^] of the *n*th layer, and *T*_*b *_is the blood temperature [°C]. Equation (1) can be solved analytically using the Laplace Transform with respect to the *z *coordinate. *T*_*n*_(*z*), which is the theoretical solution of the temperature distribution in the tissue layer *n*, is derived as follows [12]:

(2)

where

(3)

and *S*_*n*_(*z *- *z*_*n*_) is the term associated with the MMW power absorption given by the following equation:

(4)

where

(5)

(6)

Note that *Z*_0 _is the free-space wave impedance [Ω], *p *is the incident power density [W/m^2^], *α*_*n *_is the attenuation constant of the *n*-th layer [m^-1^], *β*_*n *_is the phase constant of the *n*-th layer [rad/m], *T*_*En *_is the transmission coefficient from layer *n- *1 to *n*, and *R*_*En *_is the reflection coefficient from layer *n*+1 to *n*.

Although analytically exact, solution (2) is difficult to apply to a parametric study. For this reason, a simpler analytical solution is desirable. Since the MMW power absorption is confined within the skin surface, the absorbed power can be derived from a simpler analytical solution for the one-layer (skin only) model. This physical observation can be used for an approximate solution [12], and so the body surface temperature elevation *ΔT*_3*L *_due to MMW exposure in the three-layer model can be written as follows:

(7)

(8)

where the transmission power at the air-skin interface is given by

(9)

with

(10)

and *h *is the heat transfer coefficient [W/m^2 ^°C], *Z*_*n *_is the wave impedance [Ω], *ε*_*r*1 _is the relative permittivity in the skin tissue, and *ε*_0 _is the permittivity in the vacuum [F/m].

### Approximate expressions for temperature distributions in the skin tissue

Temperature distribution *T*_1_(*z*_s_)_B _in the skin tissue before exposure can be approximated by the following expression:

(11)

where 0 ≤ *z*_s_<*z*_2_. The subscript B refers to the condition before exposure. The temperature elevation in the skin tissue Δ*T*(*z*_s_) is approximated as follows:

(12)

Furthermore, the temperature difference, Diff. *T*(Δ*z*_s_), between *z*_s1 _and *z*_s2 _due to the continuous MMW exposure can be approximated as:

(13)

where *T*_1_(0)_B _is the body surface temperature [°C] before exposure and *T*_*Air *_is the air temperature [°C].

It is difficult to investigate the effect of the dielectric permittivities on skin heating (i.e., temperature elevation and temperature difference that may cause the warm sensation, as noted previously) using the above expressions because the terms associated with the dielectric permittivities are not independent. Therefore, further approximate expressions for the temperature distributions must be derived to investigate the effect of the dielectric permittivities on skin heating. The attenuation constant of skin tissue, *α*_1 _[1/m], ranges from 1,000 to 4,000 in the MMW region, and therefore, 1/*α*_1_^2 ^may be approximately equal to zero in the equations of body surface temperature elevation (7), temperature elevation distribution in the skin tissue (12), and temperature difference in the skin tissue (13). These equations can also be approximated by the following expressions:

(14)

(15)

(16)

### Thermal, morphological, and electrical parameters

The biological parameters for our present calculations were chosen from the literature [15-22], and are listed in Table 1. The rationale for these choices is discussed in [12]. The conditions ("max" and "min"), in which the maximum and minimum body surface temperature elevations are observed, were determined in [12]. In addition, the set of commonly used parameters are named "typical" [20,21,23].

**Table 1:** Thermal constants and tissue thicknesses in the analytical model ("max"-"typical"-"min").

	***h *(surface)**	***b*_*n*_**	***κ*_*n*_**	***z*_*n*+1_- *z*_*n*_[mm]**
skin(*n *= 1)	1-10-15	6800-9100-9100	0.26-0.42-0.50	0.7-1.0-1.3
fat (*n *= 2)	-	800-1700-2100	0.20-0.25-0.27	6.3-3.5-0.7
Muscle (*n *= 3)	-	1300-2700-3500	0.43-0.50-0.55	53.0-55.5-58.0

Detailed discussions are described in [13]. The conditions, "max" and "min," in which the maximum and minimum body surface temperature elevations were observed, were determined in [12]. In addition, the set of parameters commonly used are named "typical" [20,21,23].

**Figure 2:**
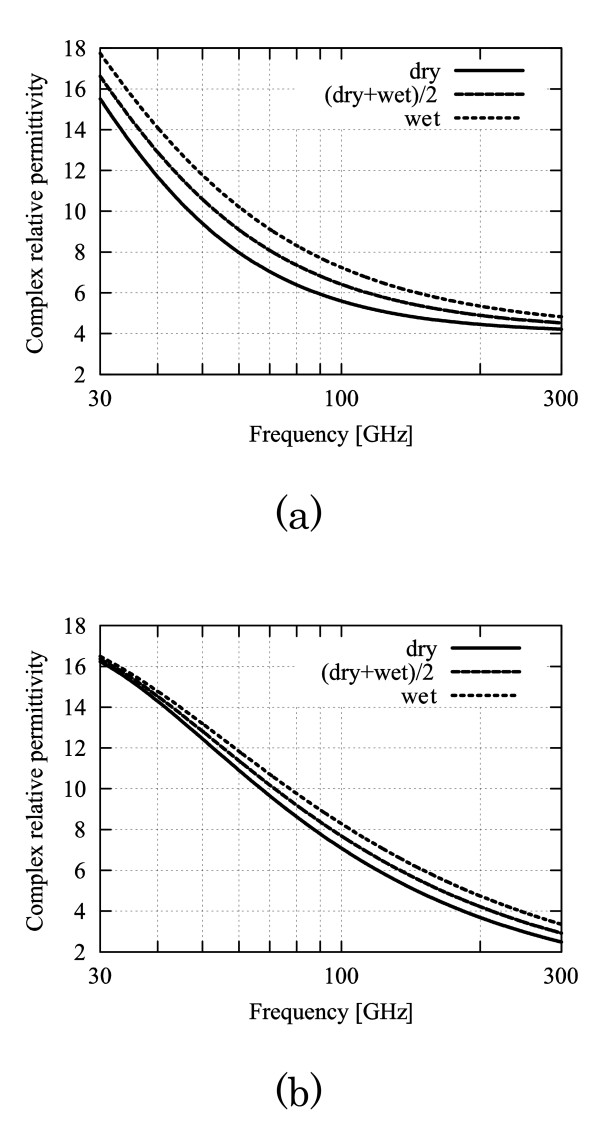
**Frequency characteristics of the complex relative dielectric permittivity in skin tissue (Gabriel *et al***. [9]). (a) Real part. (b) Imaginary part.

The dielectric permittivities of tissues were determined on the basis of [13]. The complex relative permittivity in the skin tissues "dry," "(dry + wet)/2," and "wet" (see Fig. 2) are applied to the conditions "max," "typical," and "min." In contrast, the variations in the dielectric permittivities in fat and muscle tissues are not considered because the differences in the dielectric permittivities of fat and muscle tissues have little influence on the MMW power absorption [9].

## Results and Discussion

### Approximate expressions of skin heating

**Figure 3:**
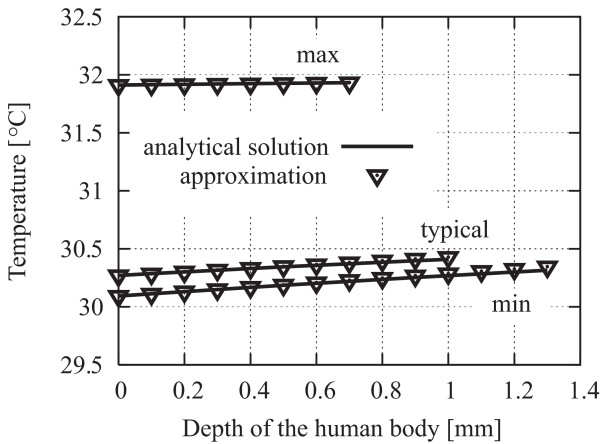
**Temperature distributions in the skin tissue before exposure**. Comparison between the results obtained from the analytical exact solution (2) and the derived approximate expression (12).

**Figure 4:**
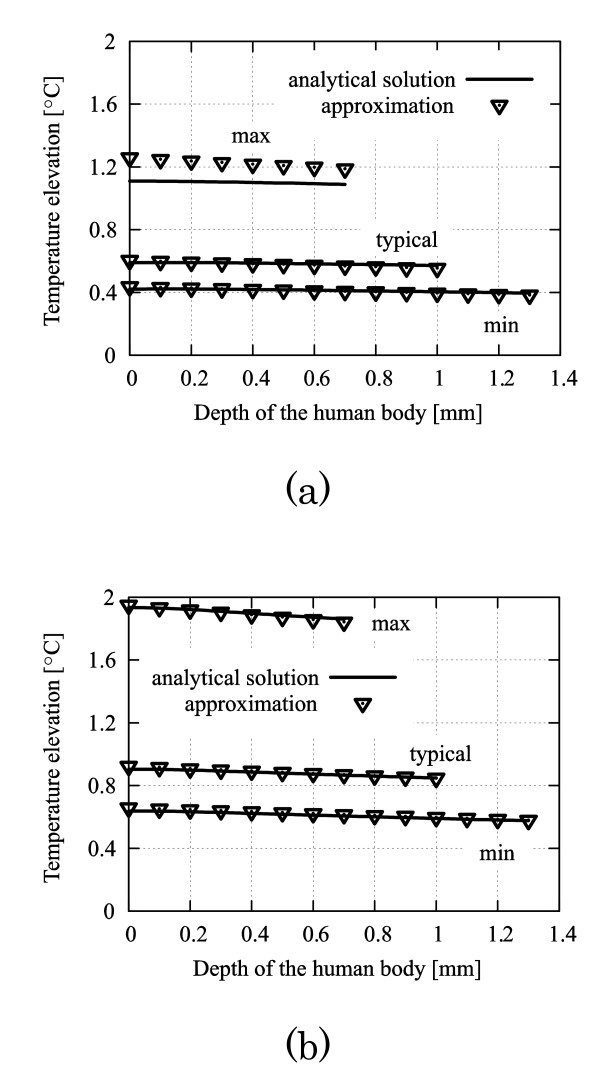
**Temperature elevations in the skin tissue due to MMW exposure**. These are calculated from the analytical exact solution (2) and approximate expression (12) under the conditions of "max", "typical" and "min" in Table 1. The incident power density *p *is 50 W/m^2^. (a) 30 GHz (b) 300 GHz

The temperature distributions in the skin tissue before exposure using an analytical exact solution (2) and its approximation (11) are shown in Fig. 3. Good agreement is shown between the analytical solution and the approximation, and relative errors are at most 1%. The temperature elevation in the skin tissue due to MMW exposure at (a) 30 GHz and (b) 300 GHz using the analytical exact solution (2) and its approximation (15) are shown in Fig. 4. The incident power density of MMW was chosen as 50 W/m^2^. As seen from Figs. 4(a) and (b), the errors attributed to the approximation increase as the frequency decreases. This tendency may be due to the increase in penetration depth of the skin tissue. The relative errors of the approximations are at most 15% under the worst condition, such as thin skin thickness and lower-frequency (30 GHz) illumination. The validity of our approximation is confirmed, and the temperature distributions before the exposure and the temperature elevations in the skin tissue due to continuous exposure calculated by the approximate expressions (11) and (15) are in satisfactory agreement with those from the analytical solution (2). Therefore, one can utilize the derived approximated formula (15) and (16) for the analytical evaluation of the effect of the dielectric permittivities on skin heating.

Let us further discuss the approximate expressions for the temperature distributions in the skin tissue. As is evident from equations (15) and (16), the term associated with dielectric permittivities *Z*_0_*pσ*_1_|*T*_*E*10_|^2^/2*α*_1 _is independent of the morphological and thermal parameters, and this term is the only factor of skin heating. Assuming that forward propagation is the dominant contribution in the skin layer, as discussed above, the distribution of the specific absorption rate (SAR [W/kg]) in the skin is derived as

(17)

where *ρ*_1 _[kg/m^3^] is the mass density of the skin tissue. Then, the total absorption power in the skin tissue due to MMW exposure *P*_*t *_is expressed as

(18)

For values of *z*_2 _shown in Table 1, *α*_1_*z*_2 _*>>α*_1_*z*_1 _= 0 in the MMW region. Therefore, the upper integration limit can be ignored to obtain

(19)

Consequently, the temperature elevation Δ_1 _(*z*_s_) and the temperature difference Diff. (Δ*z*_s_) at the location where the warm sensation occurs, correlates only with the total absorption power in the skin. In other words, it is possible to evaluate the effect of the dielectric permittivities on skin heating by the total absorption power in the skin layer per unit area rather than the incident power density or SAR values per unit area at the body surface. Note that the total absorption power per unit area in the skin corresponds to the total energy absorbed per unit area in the skin since we consider the temperature elevation in the thermal steady state.

It is worth comparing our finding in MMW with the corresponding results in microwave frequencies. For frequencies up to 3 GHz, the peak 10 g or mass-averaged SAR is used as a metric in the IEEE standards [24]. One of the rationales for this is the correlation between the 10-g averaged SAR and local temperature elevation. However, such an indicator may be inappropriate for the MMW region, because of the small penetration depth. Our analytical formula shows that the temperature elevation in skin can be estimated using the total power absorbed in the skin, instead of the mass-averaged SAR used for microwaves.

### Effects of electrical parameters on skin heating

The total absorption power is determined by the electric conductivity *σ*_1 _in the skin tissue, the attenuation constant *α*_1 _in the skin tissue, and the transmission coefficient *T*_*E*10 _at the air-skin interface, all of which are related to the dielectric permittivities. Among these parameters, *T*_*E*10 _is the most dominant factor. Therefore, the effects of the dielectric permittivities on skin heating can be investigated by using *T*_*E*10_. Because biological tissues contain a certain amount of water, which exhibits a frequency dispersive nature in the MMW region, the relative dielectric permittivity of biological tissues can be adequately approximated by a Debye-type formula [10,11]:

(20)

where *ω *[rad/s] is the angular frequency, *τ *[s] is the relaxation time, *ε*_8 _is the relative permittivity at *ωτ *>> 1, *ε*_*s *_(>*ε*_8_) is the static relative permittivity (at *ωτ *<< 1), and *σ*_*s *_[S/m] is the static conductivity. The effects of *ε*_*s*_, *τ*, and *σ*_*s *_in equation (20) on skin heating are especially considered. It is clear that the relative permittivities *ε*' and *ε *" increase with *ε*_*s *_and *σ*_*s*_. The partial derivatives of *ε*' and *ε*" with respect to *τ *are given as follows:

(21)

(22)

since 1 < (*ωτ*)^2 ^for the MMW region [9-11]. Accordingly, *ε*' and *ε*" decrease monotonically. As seen in equation (9), the complex relative permittivity *ε*_r_* is included in the denominator of *T*_*E*10_. Thus, skin heating increases as relaxation time *τ *increases.

**Figure 5:**
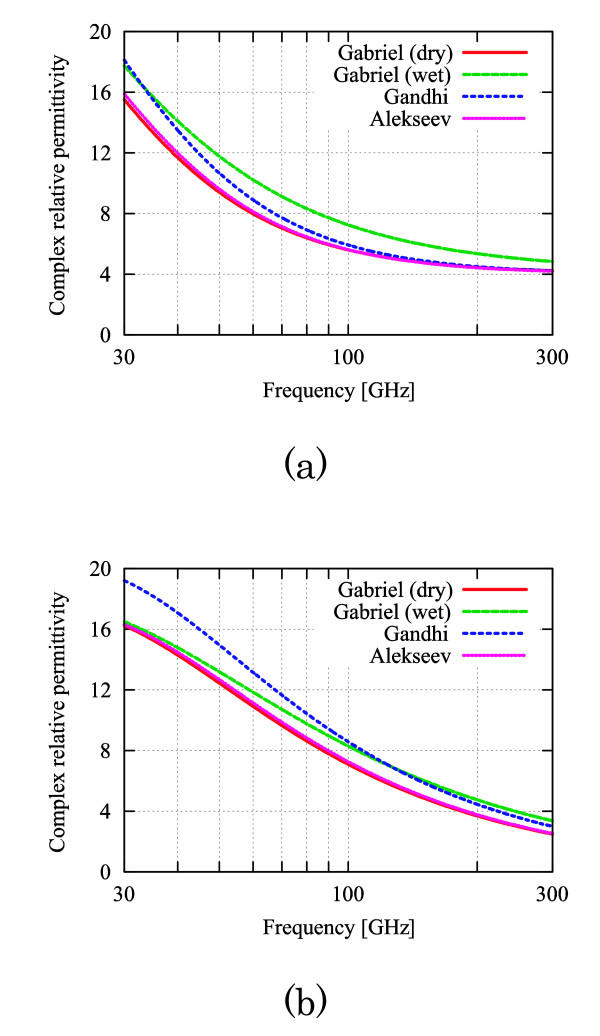
**Frequency characteristics of the dielectric permittivities in skin tissue **[9-11]. (a) Real part, (b) Imaginary part of the relative dielectric permittivity.

**Figure 6:**
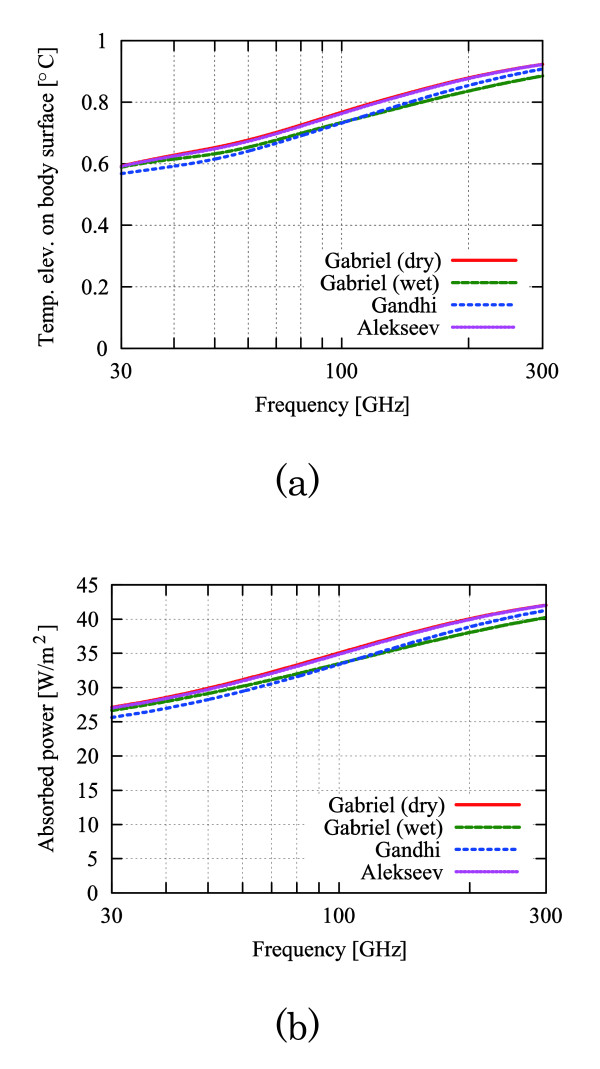
**Frequency characteristics of (a) body surface temperature elevations and (b) total absorption power**. These are calculated from the analytical solution (2) and *Z*_0_*pσ*_1_|*T*_*E*10_|^2^/2*α*_1_. The power density *p *is 50 W/m^2^, the parameter condition is "typical" in Table 1, and the dielectric permittivities in Figure 5 are from [9-11].

Let us now discuss some parameters of the effect of dielectric permittivities on the temperature elevation. Frequency dispersion characteristics of dielectric permittivities were reported in [9,13,14] and used to calculate the body surface temperature elevations and absorbed powers. These values are shown in Figs. 5 and 6, respectively. Cases exist in which the dielectric permittivities are as much as 35% larger than the values for the 'dry' skin reported by Gabriel [13]. In contrast, the variations in body surface temperature elevations and absorbed powers were less than 6% from the results using "Gabriel (dry)." The same conclusion was drawn for the temperature difference in skin tissue for other thermal and morphological parameters, because these parameters are independent of the dielectric permittivities (see equations (15) and (16)).

Both the temperature elevation Δ_1 _(*z*_*s*_) (15) and the temperature difference Diff. (Δ*z*_*s*_) (16) are found to increase monotonically with the frequency for the following reasons. The complex relative permittivity in the denominator of *T*_*E*10 _decreases monotonically with the increase in frequency (see Fig. 2). Then, the total absorption power increases with the frequency, which is attributed to the increase in transmission coefficient TE_10_. From Fig. 6(a), it is also confirmed that the body surface temperature elevations increase monotonically with the frequency.

The above results show that the maximum temperature elevation on the body surface is approximately 1.6 times the minimum value, although the body surface temperature elevation for thermal constants and tissue thicknesses varies approximately by 3 times [12]. Therefore, the effects of the dielectric permittivities on skin heating are less than those of the thermal constants and tissue thicknesses.

## Conclusion

This present study investigated the effects of dielectric permittivities on the temperature elevation and temperature difference in the thermal steady state in the skin layer of a one-dimensional three-layer model (skin, fat, muscle) for MMW exposure. Approximate expressions were derived around the location near the warm sensation receptors from the analytical solution for BHE. The derived expressions exhibit clearly that the dielectric permittivity is independent from morphological and thermal parameters. The temperature distributions in the skin tissue by our approximate expressions agree well with those derived from the analytical solution, and the relative errors are at most 15%.

Using our approximate expressions, it is shown that local skin heating can be correlated with total absorption power in the skin per unit area rather than that of the incident power density or that of SAR values per unit area at the body surface. Note that the total absorption power per unit area in the skin corresponds to the total energy absorbed per unit area in the skin since we consider the temperature elevation in the thermal steady state. Therefore, the effects of the dielectric permittivities on skin heating can be evaluated by the total absorption power in the skin tissue only. This measure is similar to the mass-averaged SAR used as a metric in the safety guidelines for microwaves [24]. The primary difference is attributed to the penetration depth of electromagnetic waves, resulting in a different SAR averaging region.

Debye-type approximation was introduced to consider the nature of the frequency dispersion of the tissue's dielectric permittivities, and the parametric study was conducted to see the effect of the dielectric permittivities on skin heating. The effects of the dielectric permittivities on skin heating are concluded to be less than those due to the thermal constants and tissue thicknesses.

Future work is to discuss the variability of MMW thermal sensation based on the finding in the present study.

## Competing interests

The authors declare that they have no competing interests.

## Authors' contributions

AK has made contributions to the conception, analysis, and interpretation and has been involved in drafting the manuscript. AH has been involved in thermal modeling and its interpretation and revising the manuscript for critically important intellectual content. SW and HS have supervised this project and have been involved in revising the manuscript. All authors have read and approved the final manuscript.
